# Performance Enhancement of Ionic Polymer-Metal Composite Actuators with Polyethylene Oxide

**DOI:** 10.3390/polym14010080

**Published:** 2021-12-26

**Authors:** Dongxu Zhao, Jie Ru, Tong Wang, Yanjie Wang, Longfei Chang

**Affiliations:** 1 College of Mechanical and Electrical Engineering, Inner Mongolia Agricultural University, Hohhot 010018, China; dxzhao611@imau.edu.cn; 2 Key Laboratory of Green and Precise Synthetic Chemistry and Applications, Ministry of Education, School of Chemistry and Materials Science, Huaibei Normal University, Huaibei 235000, China; 3 College of Mechanical and Electrical Engineering, Zhengzhou University of Light Industry, Zhengzhou 450002, China; 2009039@zzuli.edu.cn; 4 Jiangsu Key Laboratory of Special Robot Technology, Hohai University—Changzhou, Changzhou 213022, China; yjwang@hhu.edu.cn; 5 Anhui Province Key Laboratory of Aerospace Structural Parts Forming Technology and Equipment, Hefei University of Technology, Hefei 230009, China; feny.clf@hfut.edu.cn

**Keywords:** ionic polymer-metal composite, polyethylene oxide, water content, polyethylene oxide content, electromechanical properties

## Abstract

Current ionic polymer-metal composite (IPMC) always proves inadequate in terms of large attenuation and short working time in air due to water leakage. To address this problem, a feasible and effective solution was proposed in this study to enhance IPMC performance operating in air by doping polyethylene oxide (PEO) with superior water retention capacity into Nafion membrane. The investigation of physical characteristics of membranes blended with varying PEO contents revealed that PEO/Nafion membrane with 20 wt% PEO exhibited a homogeneous internal structure and a high water uptake ratio. At the same time, influences of PEO contents on electromechanical properties of IPMCs were studied, showing that the IPMCs with 20 wt% PEO presented the largest peak-to-peak displacement, the highest volumetric work density, and prolonged stable working time. It was demonstrated that doping PEO reinforced electromechanical performances and restrained displacement attenuation of the resultant IPMC.

## 1. Introduction

Tremendous efforts have been made to develop ionic polymer-metal composites (IPMCs) as soft actuators based on their inherent flexibility, light weight, and biological compatibility [1,2,3,4]. IPMCs, typically composed of polymer electrolyte sandwiched between noble metal electrodes, show large deformation with fast response at relatively low electrical excitation (usually less than 10 V) [5,6]. Because of these advantages, IPMC actuators are drawing increasing interest for applications in bionic robotics, biomedical fields, and artificial muscles [7,8].

**Figure 1:**
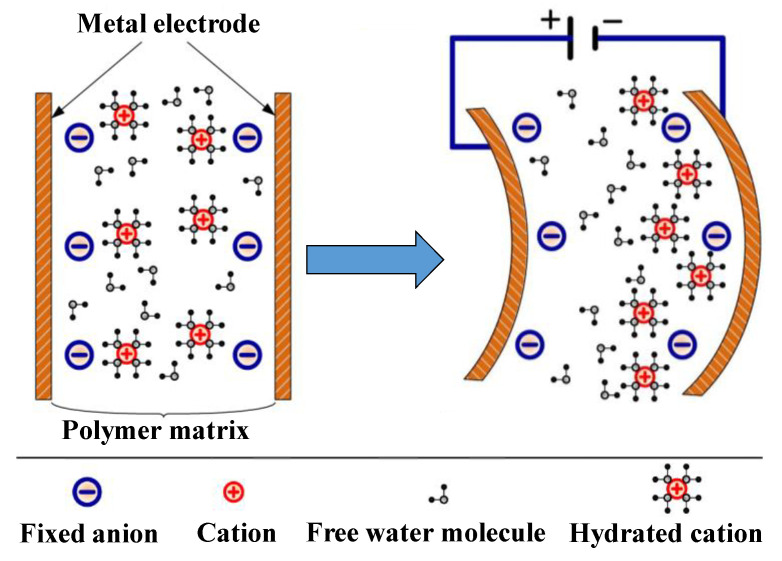
Illustration for ionic migration and deformation of IPMC.

In water-based IPMC actuators with appropriate solvent contents, water molecules bounded with counter-ions would migrate quickly to the cathode by applying an electric field between metal electrodes [9]. The migration and redistribution of water molecules contribute to the bending deformation of IPMC, as illustrated in Figure 1 [10]. It is obvious that water molecules are crucial to the actuation performance of IPMCs [11,12,13].

However, water molecules would leak from cracks in metal electrodes because of electrolysis as well as the evaporation of water when operated in air, which would significantly influence the actuation process and reduce the lifetimes of water-based IPMC actuators [14]. Therefore, increasing studies have been carried out to improve the cycling life of IPMC actuators with stable performance. Many kinds of polymer materials such as polydimethylsiloxane (PDMS), dielectric gel, and parylene were employed to encapsulate IPMC in order to reduce water leakage [15,16,17]. Although these hydrophobic coatings can help achieve a longer working time in air, the bending deformation was restrained due to this additional load. Chung et al. fabricated IPMC actuators with silver (Ag) sheet coated with a Ag nano-powders membrane [18]. As the good adhesion between the Ag electrodes and Ag-Nafion membrane can effectively prevent cracks of the actuator, the loss of water was significantly reduced and the IPMC actuator presented large displacement. Nevertheless, the high elastic modulus of the electrode still limited the blocking force of the IPMC actuator to less than 2.2 mN under 3 V. Moreover, in order to achieve stable actuation performance of IPMC, water was considered to be replaced by high boiling point solvents, such as ionic liquid and ethylene glycol (EG) [19,20]. However, the response speed of IPMC became rather low because of the high viscosity of the solvent. Polyethylene oxide (PEO) is a promising candidate for water retention material due to its unique ether-oxygen bond (-C-C-O-C-C-O-) structure and hydrogen properties [21,22,23,24,25]. These features are in favor of the formation of consecutive ion migration microchannels inside the membrane doped with PEO [26,27,28].

Herein, a new low-cost IPMC actuator with Nafion doped with PEO asmatrix was developed to have long stable working time in air and improved actuation performance. This PEO/Nafion-IPMC actuator can not only promote migration of hydrated cations, but also can increase the water content because of its consecutive microchannels. A series of evaluative methods are employed for morphology, water-retaining capacity, ionic exchange capacity (IEC), electromechanical properties of the PEO/Nafion membrane, and resultant IPMC. Strikingly, PEO/Nafion-IPMC actuator shows optimal electromechanical performances and a long stable lifetime in air, which is expected to be applied to bionic robotics at a low cost.

## 2. Experiments

### 2.1. Experimental Materials

The Nafion solution (DE-520, ~5.0 wt%) was obtained from DuPont Company (Wilmington, DE, USA). PEO, Pd(NH_3_)_4_Cl_2_, NaBH_4_, N_2_H_4_·H_2_O, and ethylene glycol (EG) was purchased from Sigma-Aldrich (St. Louis, MO, USA), with the PEO having a viscosity-average molecular weight of 200 k. All of the reagents were analytically pure and used without further purification.

### 2.2. Preparation of PEO/Nafion Membrane

A certain amount of PEO, EG, and DI water was added into a round-bottom flask and stirred for 2 h; after that, Nafion solution was poured into the flask and stirred for another 4 h. The uniform mixture was transferred into a poly(dimethylsiloxane) (PDMS, Sylgad184) container (40 mm × 60 mm × 40 mm) and treated at 70 °C in an oven to form PEO/Nafion membrane. Then the membrane was processed at 100 °C and 120 °C for 2 h, respectively, followed by annealing treatment at 150 °C for 0.5 h. The blending membranes were obtained with a PEO weight fraction of 0 to 30 wt%. The sizes of PEO/Nafion membranes were 60 mm × 40 mm × (290 ± 10) μm under dry state. The composition of the casting solutions and membranes is summarized in Table 1.

### 2.3. Fabrication of IPMCs

The PEO/Nafion-IPMCs were prepared via the impregnation-reduction method, which has been described in detail in the [10]. First, the PEO/Nafion membranes were roughened by sandpaper (class 1200) and boiled in acid solution (2 mol/L HCl) and the deionized water, respectively. Then the palladium electrode was preliminarily painted on both sides of the membrane for 3 times and then chemically plated for 2 times. Finally, the IPMCs were obtained and soaked in 0.2 mol/L HCl for 2 times (2 h each time) to exchange the cations in the membrane with H^+^ cation. In order to acquire the actuation capability, the IPMCs were immersed in 0.2 mol/L NaOH solution for 2 times (2 h each time) to absorb Na^+^ as mobile ions. Later, IPMCs were cut into small pieces with 35 mm × 5 mm × (320 ± 10) μm under hydrated conditions for testing the performances. Images of the samples are shown in Supplementary Materials Figure S2 (Supporting Information).

### 2.4. Characterizations

#### 2.4.1. Characterizations of PEO/Nafion Membranes

In order to reveal the regularity inside the PEO/Nafion membrane, a field emission scanning electron microscope (FE-SEM, Zeiss GenimiSEM 500) was used to observe the cross-sectional morphologies of IPMC samples.

Aiming to detect the water-uptake ratio (WUR), the samples were placed in DI water for 24 h; before the saturated samples were weighed and recorded as *M_wet_* (g), water on the surface was carefully wiped off with filter paper. Next, the mass of the samples under dry condition was recorded as *M_dry_* (g), immediately after drying at 100 °C for at least 24 h. The WUR (*w*) of each sample was calculated according to the following Equation (1):

(1) $$w\;\left(\%\right)=\frac{M_{wet}-M_{dry}}{M_{dry}}\times 100\%,$$

The ionic exchange capacity (*IEC*) defined as Equation (2) was investigated via the acid-base titration method with NaOH (aq) and HCl (aq), which is described in detail in the Reference [10].

(2) $$IEC=\frac{V_{\mathrm{NaOH}}\times N_{\mathrm{NaOH}}}{m_{membrane}}\left(meq/g\right),\;$$

where *V*_NaOH_ and *N*_NaOH_ are the volume (mL) and the normality (mol) of NaOH (aq), respectively, and *m_membrane_* is the mass (g) of the membrane. To reduce experimental error, at least three sets of experiments were carried out.

#### 2.4.2. Electromechanical Test of IPMCs

The equivalent stiffness of the IPMC sample was characterized by adopting the free oscillation attenuation method [6]. Each specimen was measured in the wet state within 2 min. The equivalent stiffness (*E*) can be described by Equation (3), which is derived from the Euler–Bernoulli beam theory.

(3) $$E={\left(\frac{2\pi }{3.52}\right)}^{2}\frac{mf^{2}l^{3}}{I}=3.87\frac{\pi^{2}mf^{2}l^{3}}{ht^{3}},\;$$

where *m* is the quantity (kg) of the free end in the configuration, *f* is the natural frequency (Hz), *I* is a moment of inertia (m^4^), *d* is the measuring distance (m), *l*, *h*, and *t* are the free length (m), the width (m) and the thickness (m) of the specimen, respectively.

Actuation performances were represented by detecting the deformation and blocking force of IPMCs with the same experimental facilities described in the Reference [10]. The test platform is shown in Supplementary Materials Figure S1 (Supporting Information) [5]. The sample was fastened by a gold-plated copper clamp on one side with a free length of 30 mm. The measuring points of all the samples were 20 mm away from the fastened end. All the experiments were performed in the air. At least three parallel specimens per group were tested, and the relative standard deviations of each group were no more than 20%.

## 3. Results and Discussion

### 3.1. Influences of PEO Content on the Properties of the PEO/Nafion Membranes

**Figure 2:**
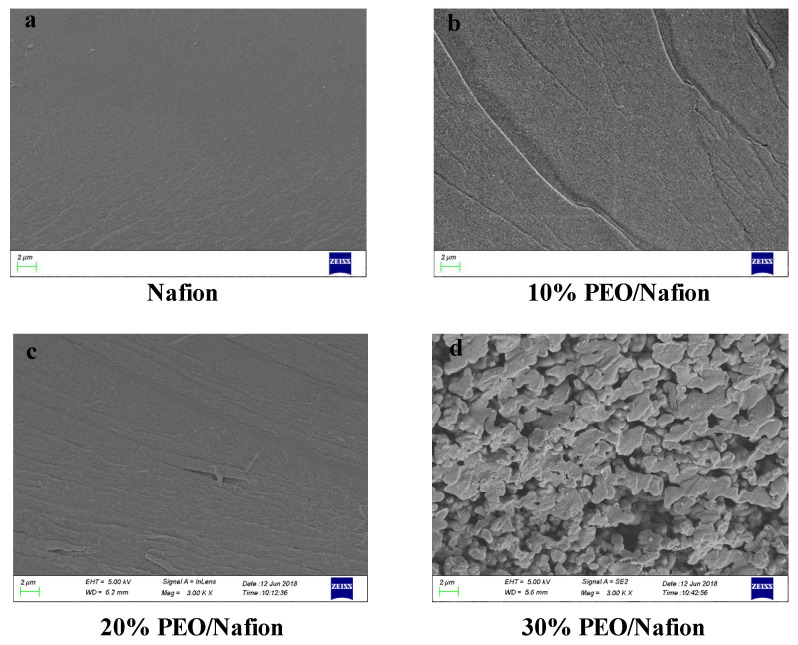
The cross-sections of Nafion membranes with PEO contents of (**a**) 0 wt%, (**b**) 10 wt%, (**c**) 20 wt% and (**d**) 30 wt%, respectively.

Figure 2 shows cross-sectional SEM morphologies of the Nafion membranes with PEO of varying content. Without PEO added to the Nafion solution, the cross-section of the membrane was typically smooth (Figure 2a). It can be seen from Figure 2b,c that internal morphologies of the membranes blended with PEO content of 10 wt% and 20 wt% were basically smooth with only several wrinkles, which were rather similar to that of the pure Nafion membrane. It was this homogeneous internal structure that contributed to the excellent electromechanical performance for the resultant IPMC. Whereas PEO content increased, morphologies of the blended membranes changed significantly. As PEO content rose to 30 wt% (Figure 2d), a large number of holes appeared in the PEO/Nafion membranes, which might be explained by phase separation with the Nafion matrix. It indicated that compact and uniform internal structure could not form in the membrane with excess PEO, which might be a disadvantage for electromechanical performances of resultant IPMCs.

**Figure 3:**
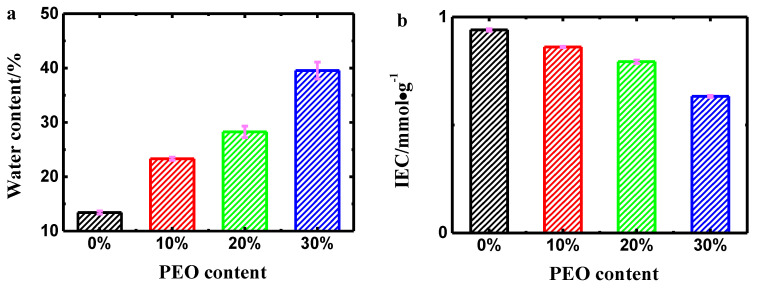
WUR (**a**) and IEC (**b**) of Nafion membranes with varying PEO contents.

According to the Grotthus ‘hopping’ theory and vehicle mechanisms, WUR and IEC are essential factors dramatically affecting the hydrated cation migration in Nafion membranes in terms of mechanical strength and actuation property of the resultant IPMCs [29,30]. Figure 3a presents that WUR values of the PEO/Nafion membranes were significantly increased with the addition of PEO. This result can be explained by the fact that water retention capacity of PEO is superior to that of Nafion. In addition, the blended membrane with 30 wt% PEO exhibited a high WUP (39.54%), which was nearly 3 times that of the pure Nafion membrane (13.40%). Therefore, the resultant PEO/Nafion-IPMCs could present better electromechanical properties. The IEC of blended membranes gradually decreased as the PEO content rose (Figure 3b). The average IEC value of the specimen with 30 wt% PEO content reduced to 0.63 mmol·g^−1^, which was about 67 percent of the value of the pure Nafion membrane (0.94 mmol·g^−1^). This result can be explained by that PEO has little capacity for ion exchange. Therefore, that would reduce the IEC of the membrane by replacing Nafion with PEO. However, this result might hamper the electromechanical property of resultant IPMCs.

### 3.2. Influences of PEO Content on the Properties of the PEO/Nafion-IPMCs

**Figure 4:**
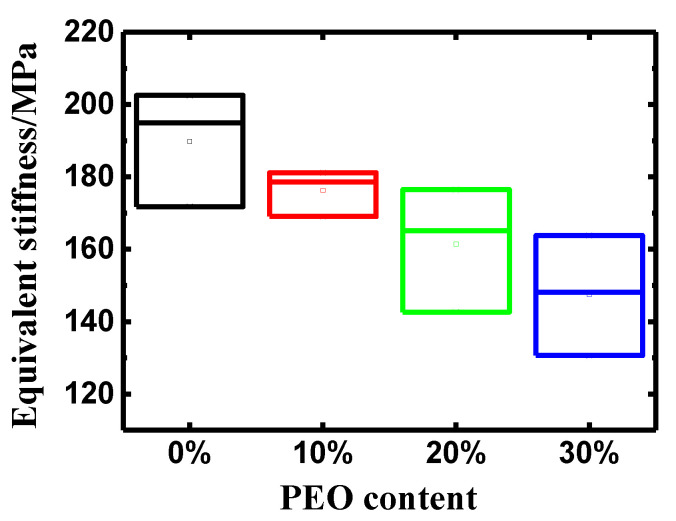
Equivalent stiffness of PEO/Nafion-IPMC with varying PEO contents.

As a principal element of the actuation performance, equivalent stiffness of IPMCs with varying PEO contents was detected and is shown in Figure 4. It was observed that the equivalent stiffness of IPMCs dropped with the addition of PEO, with a similar tendency to that of IEC in PEO/Nafion membranes. The IPMCs with 30 wt% PEO presented the lowest equivalent stiffness in all specimens (147.49 MPa), which was approximately 78 percent of the value of the pure Nafion-IPMC (189.76 MPa). This result would be unfavorable for improving the blocking force of IPMCs, but helpful to bending deformation.

**Figure 5:**
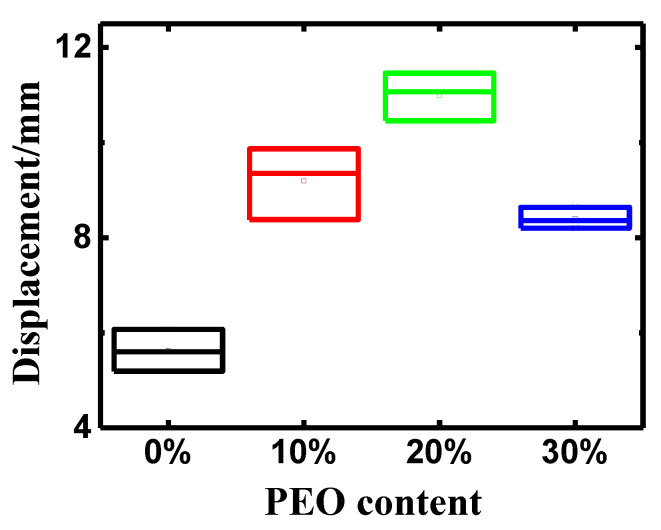
Peak-to-peak displacement of PEO/Nafion-IPMC with varying PEO contents.

**Figure 6:**
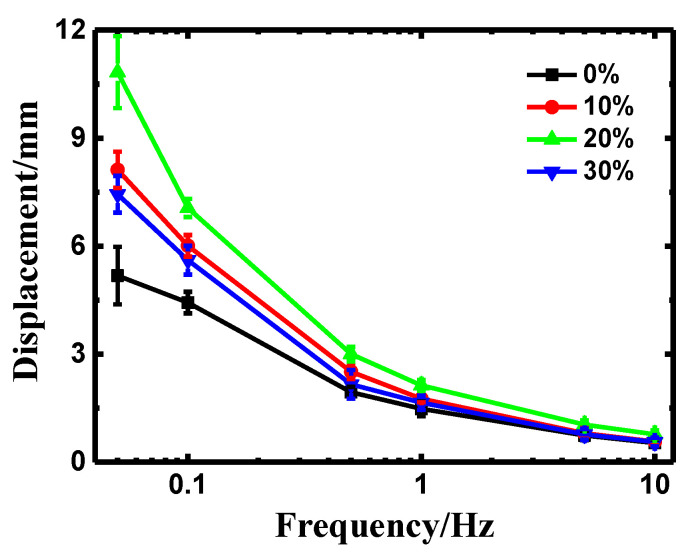
Amplitude-frequency response of PEO/Nafion-IPMC with varying PEO contents.

The deformation performance is one of the most important factors in evaluating the electromechanical performance of IPMC. Figure 5 records the peak-to-peak displacement of the PEO/Nafion-IPMC actuators under DC voltage with the amplitude of 2 V within 25 s. Obviously, doping PEO in the Nafion membrane dramatically affected the displacement property. Figure 6 reveals that the peak-to-peak displacements of all the PEO/Nafion-IPMCs were larger than those of the pure Nafion-IPMC due to higher WUR and lower equivalent stiffness of the PEO/Nafion membrane. It can be noticed that the average peak-to-peak displacement of PEO/Nafion-IPMC with 20 wt% PEO reached up to 11.00 mm, which was almost twice as large as that of the pure Nafion-IPMC (5.62 mm). This result indicated that the IPMC with a PEO content of 20 wt% presented the optimal deformation property from a comprehensive effect of appropriate WUR, IEC, equivalent stiffness, and homogeneous internal structure. However, a further increase in PEO content resulted in decreasing the peak-to-peak displacement of PEO/Nafion-IPMC, which might be due to complicated aspects, such as low IEC, a lack of counterions, or loose internal structure.

Figure 6 exhibits amplitude-frequency response of the deformation of the IPMCs under sinusoidal voltage with an amplitude of 2 V. It was confirmed that doping PEO could enormously enhance the deformation property of the resultant IPMCs, compared with the pure Nafion-IPMC. It also can be seen that the displacement of the specimens fell sharply while the excitation frequency rose. This result can be explained that counter ions in IPMC failed to accommodate the voltage variation [31]. Furthermore, these results followed the same tendency as the peak-to-peak displacement, which confirmed that the PEO/Nafion-IPMCs with PEO content of 20 wt% had the optimal effect on the deformation performances.

**Figure 7:**
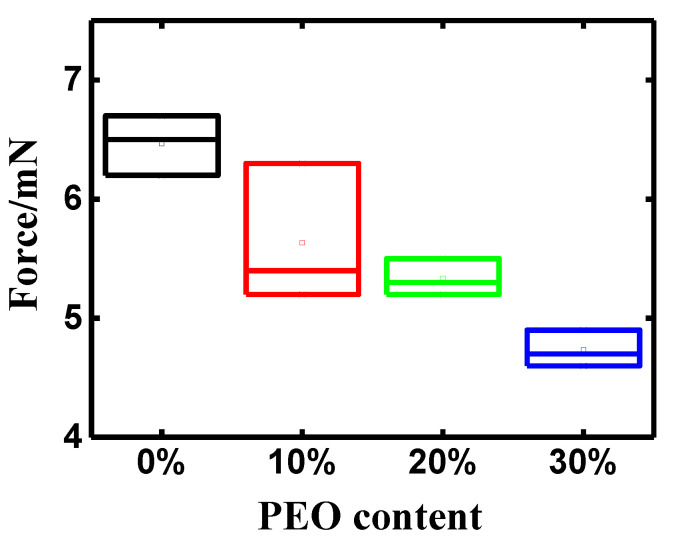
The peak-to-peak blocking force of PEO/Nafion-IPMC with varying PEO contents.

The peak-to-peak blocking forces of the IPMCs with PEO contents of 0 wt%, 10 wt%, 20 wt%, and 30 wt% are presented in Figure 7. It can be seen that the blocking force, unlike the displacement of PEO/Nafion-IPMC, declined sharply with the addition of PEO in the Nafion matrix in comparison with the pure Nafion-IPMC. The average peak-to-peak blocking force of the pure Nafion-IPMC was 6.47 mN, while that of the PEO/Nafion-IPMC with 30 wt% PEO was as low as 4.73 mN. The results may be due to such impacts as the lower IEC, fewer counter ions, and significantly reduced equivalent stiffness with the addition of PEO.

The displacement and blocking force of IPMCs varied dramatically according to the sample dimensions. The volumetric work density (*W_D_*) was calculated from the performance parameters and size of IPMCs by using the following Equation [32]:

(4) $$W_{D}=\frac{2d}{3ht}\frac{F\delta }{d^{2}+\delta^{2}},\;$$

where *d* is the measuring distance, *h* is the width, *t* is the thickness, *δ* and *F* are the peak-to-peak displacement and blocking force under 2 V DC voltage of the IPMCs, respectively. The volumetric work density values of PEO/Nafion-IPMCs are summarized in Table 2. It can be observed that compared with the pure Nafion-IPMC, PEO/Nafion-IPMCs exhibited higher volumetric work density. Notably, the IPMC specimen with 20% PEO showed the highest volumetric work density of all, which demonstrated the optimal actuation performance. Although the volumetric work density value of the IPMC with 20 wt% was quite close to the value of the 10% sample, the former showed more advantages in higher WUP and less cost. As summarized in Supplementary Materials Table S1 (Supporting Information), the displacement and blocking force are higher than those of other types of bending actuator, at a 2 V DC voltage as well as 0.1 and 1 Hz square-wave activation for PEO/Nafion-IPMC with 20 wt% PEO [2,33,34,35,36,37]. However, the blocking force of IAEP-IPMC [2] is higher than that of PEO/Nafion-IPMC, as a result of the high-quality metal electrodes of the former actuator fabricated via the isopropanol-assisted electroless plating. Nevertheless, the PEO/Nafion-IPMC with 20 wt% PEO exhibits a large deformation and fast response, the outstanding integration of which makes the IPMC actuator a promising candidate for actuation applications.

**Figure 8:**
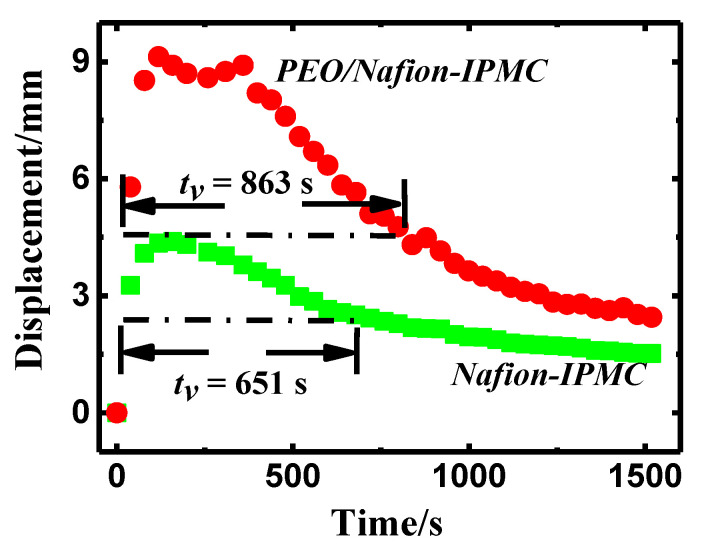
Displacement attenuation curves of IPMCs with 20 wt% PEO and pure Nafion.

In order to evaluate the stability of actuation performance over the time in air, a valid working time in air was introduced and recorded as *t_v_*, which was defined as the time when it is larger than half of the peak value. The displacement attenuation curves of IPMCs under sinusoidal voltage of 2 V amplitude and 0.1 Hz frequency, are recorded in Figure 8. It can be seen that the deformation of IPMC increased first and then decreased with time in air, due to changes in electromechanical parameters resulted from water leakage. It is evident that *t_v_* of the pure Nafion-IPMC and PEO/Nafion-IPMC with 20 wt% PEO was 651 s and 863 s, respectively, with a difference of 33% increase. Therefore, after doping PEO in the Nafion matrix, IPMCs presented enormously enhanced displacement and prolonged lifetime in air.

## 4. Conclusions

The traditional Nafion-IPMC presented smaller deformation and larger attenuation, due to water leakage and low WUR. To address these problems, a high WUR and flexible PEO/Nafion-IPMC were successfully prepared by doping PEO in the Nafion matrix. A series of methods were employed to demonstrate the PEO/Nafion membrane blended with uniform internal structure and high WUR. Importantly, it was verified that the resultant PEO/Nafion-IPMCs with 20 wt% PEO exhibited an increased peak-to-peak displacement, nearly twice that of the pure Nafion-IPMC, a remarkably enhanced volumetric work density, and a significantly prolonged valid working time in air. Although the addition of PEO caused a decrease in IEC and blocking force of the specimen, this study provides a novel and effective solution to reinforce electromechanical property and to restrain displacement attenuation.

## Figures and Tables

**Table 1:** Composition of the casting solutions for PEO/Nafion blending membranes.

PEO Content	Nafion (aq)	PEO (s)	DMF (l)	H_2_O (l)
0 wt%	26.250 g	0 g	6.3 g	0 g
10 wt%	23.625 g	0.1313 g	6.3 g	2.494 g
20 wt%	21.000 g	0.2625 g	6.3 g	4.988 g
30 wt%	18.375 g	0.3940 g	6.3 g	7.480 g

**Table 2:** Volumetric work density values of PEO/Nafion-IPMCs with varying PEO contents.

Sample	0%	10%	20%	30%
*W_D_* (KJ/m^3^)	0.60	0.87	0.88	0.69

## Data Availability

Not applicable.

## Supplementary Materials

The following supporting information can be downloaded at: https://www.mdpi.com/article/10.3390/polym14010080/s1, Figure S1: (a) Schematic of the experimental set-up. (b) Visual representation of the platform, Figure S2: Images of the IPMC samples, Table S1: Displacements and blocking forces of different bending actuators.

## References

[1] Zhou L., Fu J., He Y. (2020). A Review of 3D Printing Technologies for Soft Polymer Materials. Adv. Funct. Mater..

[2] Ma S.Q., Zhang Y.P., Liang Y.H., Ren L., Tian W.J., Ren L.Q. (2019). High-Performance Ionic-Polymer-Metal Composite: Toward Large-Deformation Fast-Response Artificial Muscles. Adv. Funct. Mater..

[3] White B.T., Long T.E. (2018). Advances in Polymeric Materials for Electromechanical Devices. Macromol. Rapid Commun..

[4] Shen Q., Stalbaum T., Minaian N., Oh I., Kim K.J. (2018). A robotic multiple-shape-memory ionic polymer-metal composite (IPMC) actuator: Modeling approach. Smart Mater. Struct..

[5] Ru J., Zhu Z., Wang Y., Chen H., Li D. (2018). Tunable actuation behavior of ionic polymer metal composite utilizing carboxylated carbon nanotube-doped Nafion matrix. RSC Adv..

[6] Zhao D., Li D., Wang Y., Chen H. (2016). Improved manufacturing technology for producing porous Nafion for high-performance ionic polymer–metal composite actuators. Smart Mater. Struct..

[7] Chen Z., Shatara S., Tan X. (2009). Modeling of Biomimetic Robotic Fish Propelled by an Ionic Polymer-Metal Composite Caudal Fin. IEEE/ASME Trans. Mechatron..

[8] Aureli M., Kopman V., Porfiri M. (2009). Free-Locomotion of Underwater Vehicles Actuated by Ionic Polymer Metal Composites. IEEE/ASME Trans. Mechatron..

[9] Zhu Z., Chang L., Takagi K., Wang Y., Chen H., Li D. (2014). Water content criterion for relaxation deformation of Nafion based ionic polymer metal composites doped with alkali cations. Appl. Phys. Lett..

[10] Wang Y., Chen H., Wang Y., Luo B., Chang L., Zhu Z., Li B. (2013). Influence of additives on the properties of casting nafion membranes and SO-based ionic polymer-Metal composite actuators. Polym. Eng. Sci..

[11] Shoji E., Hirayama D. (2007). Effects of Humidity on the Performance of Ionic Polymer-Metal Composite Actuators: Experimental Study of the Back-Relaxation of Actuators. J. Phys. Chem. B.

[12] Nemat-Nasser S., Wu Y. (2003). Comparative experimental study of ionic polymer-metal composites with different backbone ionomers and in various cation forms. J. Appl. Phys..

[13] Zhu Z., Asaka K., Chang L., Takagi K., Chen H. (2013). Physical interpretation of deformation evolvement with water content of ionic polymer-metal composite actuator. J. Appl. Phys..

[14] Guo D., Wang L., Wang X., Xiao Y., Wang C., Chen L., Ding Y. (2019). PEDOT coating enhanced electromechanical performances and prolonged stable working time of IPMC actuator. Sens. Actuators B Chem..

[15] Franklin J.W. (2003). Electromechanical Modeling of Encapsulated Ionic Polymer Transducers. Master’s Thesis.

[16] Yu C.-Y., Zhang Y.-W., Su G.-D. (2015). Reliability tests of ionic polymer metallic composites in dry air for actuator applications. Sens. Actuators A Phys..

[17] Kim S.J., Lee I.T., Lee H.-Y., Kim Y.H. (2006). Performance improvement of an ionic polymer-metal composite actuator by parylene thin film coating. Smart Mater. Struct..

[18] Chung C.-K., Fung P., Hong Y., Ju M., Lin C.-C., Wu T. (2006). A novel fabrication of ionic polymer-metal composites (IPMC) actuator with silver nano-powders. Sens. Actuators B Chem..

[19] Bennett M.D., Leo D.J. Ionic Liquids as Hyper-Stable Solvents for Ionic Polymer Transducers. Proceedings of the ASME International Mechanical Engineering Congress & Exposition.

[20] Nemat-Nasser S., Zamani S., Tor Y. (2006). Effect of solvents on the chemical and physical properties of ionic polymer-metal com-posites. J. Appl. Phys..

[21] Tant M.R., Mauritz K.A., Wilkes G.L. (1997). Ionomers: Synthesis, Structure, Properties and Applications.

[22] Thangamuthu R., Lin C. (2005). DBSA-doped PEG/SiO2 proton-conducting hybrid membranes for low-temperature fuel cell applications. Solid State Ionics.

[23] Bijay P.T., Vinod K.S. (2011). Organic-inorganic nanocomposite polymer electrolyte membranes for fuel cell applications. Prog. Polym. Sci..

[24] Kang M.-S., Lee M.-J. (2009). Anhydrous solid proton conductors based on perfluorosulfonic ionomer with polymeric solvent for polymer electrolyte fuel cell. Electrochem. Commun..

[25] Smith G.D., Bedrov D., Borodin O. (2000). Conformations and Chain Dimensions of Poly(ethylene oxide) in Aqueous Solution: A Molecular Dynamics Simulation Study. J. Am. Chem. Soc..

[26] Thangamuthu R., Lin C. (2006). Preparation of gas diffusion electrodes using PEG/SiO2 hybrid materials and the effect of their composition on microstructure of the catalyst layer and on fuel cell performance. J. Power Sources.

[27] Chang H., Lin C.W. (2003). Proton conducting membranes based on PEG/SiO2 nanocomposites for direct methanol fuel cells. J. Membr. Sci..

[28] Chang H., Thangamuthu R., Lin C. (2004). Structure–property relationships in PEG/SiO2 based proton conducting hybrid membranes—A 29Si CP/MAS solid-state NMR study. J. Membr. Sci..

[29] Wang Y.J., Chen H.L., Wang Y.Q., Zhu Z.C., Li D.C. (2014). Effect of dehydration on the mechanical and physicochemical prop-erties of gold-and palladium-ionomeric polymer-metal composite (IPMC) actuators. Electrochim. Acta.

[30] Park J., Palmre V., Hwang T., Kim K., Yim W., Bae C. (2014). Electromechanical performance and other characteristics of IPMCs fabricated with various commercially available ion exchange membranes. Smart Mater. Struct..

[31] Terasawa N., Ono N., Mukai K., Koga T., Higashi N., Asaka K. (2012). A multi-walled carbon nanotube/polymer actuator that surpasses the performance of a single-walled carbon nanotube/polymer actuator. Carbon.

[32] An Y. (2009). Mechanical and Electromechanical Performance of Ionic Polymer Metal Composite (IPMC). Ph.D. Thesis.

[33] Lee J.-W., Hong S.M., Koo C.M. (2013). High-performance polymer ionomer–ionic liquid membrane IPMC actuator. Res. Chem. Intermed..

[34] Mousavi M.S.S., Alaei A., Hasani M., Kolahdouz M., Manteghi F., Ataei F. (2018). Fabrication of ionic polymer metal composite for bio-actuation application: Sputtering and electroless plating methods. Mater. Res. Express.

[35] Ishiki A., Nabae H., Kodaira A., Suzumori K. (2020). PF-IPMC: Paper/Fabric Assisted IPMC Actuators for 3D Crafts. IEEE Robot. Autom. Lett..

[36] Luo B., Chen Z. (2017). Comparative Experimental Study on Ionic Polymer Mental Composite based on Nafion and Aquivion Membrane as Actuators. IOP Conf. Ser. Mater. Sci. Eng..

[37] Asaka K., Mukai K., Sugino T., Kiyohara K. (2013). Ionic electroactive polymer actuators based on nano-carbon electrodes. Polym. Int..

