# Supplementary material for: Performance Enhancement of Ionic Polymer-Metal Composite Actuators with Polyethylene Oxide
Source: Polymers (Basel). 2021 Dec 26;14(1):80. doi: 10.3390/polym14010080 (PMC8747705; doi:10.3390/polym14010080)
Supplement: Supplementary file 1 [file polymers-14-00080-s001.zip › polymers-1359370-supplementary.pdf]

polymers

MDPI

Supporting Information

Enhancement of Ionic Polymer-Metal Composite Actuators with Polyethylene Oxide in Air Operating

Dongxu Zhao [1,#], Jie Ru [2,#,*], Tong Wang [3], Yanjie Wang [4] and Longfei Chang [5]

[1] College of Mechanical and Electrical Engineering, Inner Mongolia Agricultural University, Hohhot 010018, People's Republic of China; dxzhao611@imau.edu.cn
[2] Institute of Mold Technology, Changzhou Vocational Institute of Mechatronic Technology, Changzhou 213164, People's Republic of China; rujie@hhu.edu.cn
[3] College of Mechanical and Electrical Engineering, Zhengzhou University of Light Industry, Zhengzhou 450002, People's Republic of China; 2009039@zzuli.edu.cn
[4] Jiangsu Key Laboratory of Special Robot Technology, Hohai University-Changzhou, Changzhou 213022, People's Republic of China; yjwang@hhu.edu.cn
[5] Anhui Province Key Lab of Aerospace Structural Parts Forming Technology and Equipment, Hefei University of Technology, Hefei 230009, People's Republic of China; feny.clf@hfut.edu.cn
[#] These authors contribute equally to this work
* Correspondence: rujie@hhu.edu.cn

1. Test platform

Figure S1 shows the test platform.

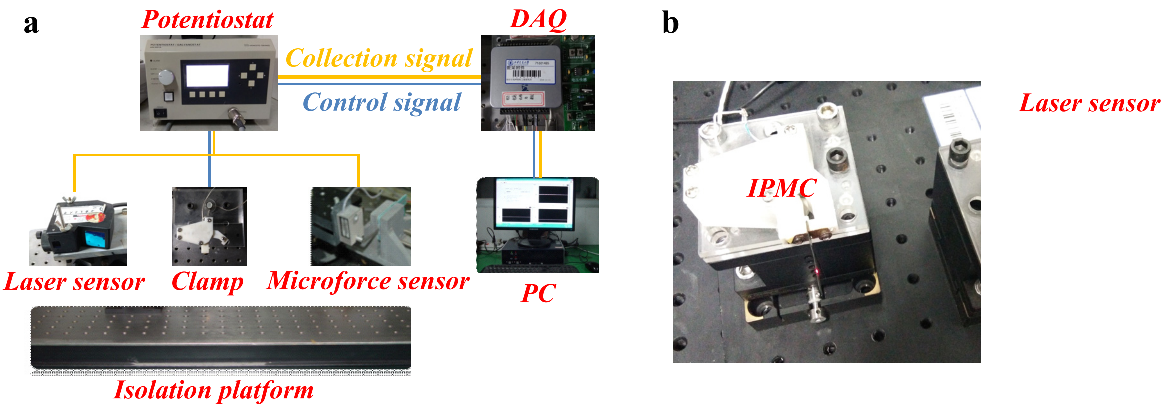

Figure S1. (**a**) Schematic of the experimental set-up. (**b**) Visual representation of the platform.

Figure S2 shows the images of the IPMC samples with different PEO contents.

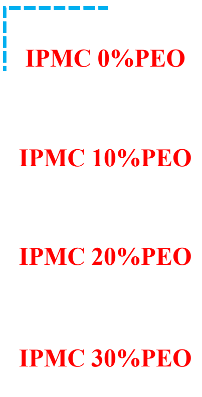

Figure S2. Images of the IPMC samples.

2. Displacement and Blocking Force

Table S1 shows the displacement and blocking force of different bending actuators under different voltages.

Table S1. Displacements and blocking forces of different bending actuators.

Name	Voltage	Frequency (Hz)	Displacemet (mm)	Blocking force (mN)	Ref[a)]
20%-PEO/Nafion-IPMC	2VDC	-	11.0	5.33	This work
	2VAC	0.1	6.01	-	
		1	1.76	-	
IAEP-IPMC	2VDC	-	7	7.4	2
SSPB actuator	2VDC	-	5.2	-	33
PS IPMC	2VDC	-	2.7	1.8	34
CEP IPMC	2VDC	-	6.5	3.1	34
Tissue-IPMC	2VDC	-	0.7	0.09	35
Kimwipe-IPMC	2VDC	-	0.4	0.03	35
Kimtowel-IPMC	2VDC	-	0.21	0.07	35
Aquivion-IPMC	2VDC	-	4.5	1.6	36
SG-SWNT actuator	2VAC	0.1	1.25	-	37
		1	0.5	-	

[a)] Reference number from manuscript.
